# Description and life-cycle of *Taenia lynciscapreoli* sp. n. (Cestoda, Cyclophyllidea)

**DOI:** 10.3897/zookeys.584.8171

**Published:** 2016-04-25

**Authors:** Voitto Haukisalmi, Sergey Konyaev, Antti Lavikainen, Marja Isomursu, Minoru Nakao

**Affiliations:** 1 Finnish Museum of Natural History Luomus, P.O. Box 17, FI–00014 University of Helsinki, Finland; 2 Institute of Systematics and Ecology of Animals SB RAS, 630091, Frunze str. 11, Novosibirsk, Russia; 3 Immunobiology Program/Department of Bacteriology and Immunology, Faculty of Medicine, P.O. Box 21, FI–00014 University of Helsinki, Finland; 4 Finnish Food Safety Authority Evira, Elektroniikkatie 3, FI–90590 Oulu, Finland; 5 Department of Parasitology, Asahikawa Medical University, Asahikawa, Hokkaido 078–8510, Japan

**Keywords:** Tapeworms, *Lynx*, *Capreolus*, *Alces*, wolf, Finland, Russia, Siberia

## Abstract

A new species of tapeworm, *Taenia lynciscapreoli* sp. n. (Cestoda, Cyclophyllidea), is described from the Eurasian lynx (*Lynx lynx*), the main definitive host, and the roe deer (*Capreolus capreolus* and *Capreolus pygargus*), the main intermediate hosts, from Finland and Russia (Siberia and the Russian Far East). The new species was found once also in the wolf (*Canis lupus*) and the Eurasian elk/moose (*Alces alces*), representing accidental definitive and intermediate hosts, respectively. The conspecificity of adult specimens and metacestodes of *Taenia lynciscapreoli* sp. n. in various host species and regions, and their distinction from related species of *Taenia*, was confirmed by partial nucleotide sequences of the mitochondrial cytochrome *c* oxidase subunit 1 gene. Morphologically, *Taenia lynciscapreoli* **sp. n.** can be separated unambiguously from all other species of *Taenia* by the shape of its large rostellar hooks, particularly the characteristically short, wide and strongly curved blade. If the large rostellar hooks are missing, *Taenia lynciscapreoli* may be separated from related species by a combination of morphological features of mature proglottids. It is suggested that *Taenia lynciscapreoli* has been present in published materials concerning the tapeworms of *Lynx lynx* and *Lynx pardinus* in Europe, but has been misidentified as *Taenia pisiformis* (Bloch, 1780). *Taenia lynciscapreoli* **sp. n.** has not been found in lynx outside the range of roe deer, suggesting a transmission pathway based on a specific predator–prey relationship. The present study applies a novel, simple approach to compare qualitative interspecific differences in the shape of rostellar hooks.

## Introduction

Morphological differences between independent species of the genus *Taenia* Linnaeus, 1758 and related genera are often limited, and it can be expected that extensive surveys based on molecular methods will reveal unknown, more or less cryptic species. In favour of this idea, at least two probable new species were recently identified in molecular phylogenetic analyses by Terefe et al. (2014) on *Taenia* spp. of the spotted hyena *Crocuta crocuta*. In addition, *Taenia arctos* Haukisalmi, Lavikainen, Laaksonen & Meri, 2011, which uses bears of the genus *Ursus* as definitive hosts (Haukisalmi et al. 2011, Lavikainen et al. 2011, Catalano et al. 2014, 2015), was originally identified as a genetically independent lineage in a cervid intermediate host (*Alces alces*; Lavikainen et al. 2010).

A recent molecular phylogenetic study on *Taenia* spp. in the Eurasian lynx (*Lynx lynx*) from Finland revealed a genetic lineage, which could not be associated with any known species based on sequence data (Lavikainen et al. 2013). In addition, the rostellar hooks of the unknown lineage were shorter than in any *Taenia* species parasitizing felids in the Holarctic region, strongly suggesting presence of a new species. Phylogenetically, the unknown *Taenia* sp. was closely related to *Taenia hydatigena* Pallas, 1766 and *Taenia regis* Baer, 1923 from canids and felids (*Panthera* spp.), respectively. At that point, the intermediate hosts of the putative new species were unknown.

Since the report by Lavikainen et al. (2013), we have been able to collect additional molecular and morphological data of the unknown species from felids and cervids, which evidently represent the main definitive and intermediate hosts, respectively, of the new lineage. We here present the new data and describe the previously unknown species as *Taenia lynciscapreoli* sp. n.

## Material and methods

The material used in the description of the new species consisted of 14 adult specimens: seven from *Lynx lynx* from Finland (four host individuals), five from the same host species from the Russian Federation (four host individuals), and two from the wolf (*Canis lupus*) from Russia (one host individual).

In addition, 11 metacestodes (cysticerci) were examined to characterize the rostellar hooks of the new species: two specimens from the European roe deer *Capreolus capreolus* and five specimens from the Eurasian elk/moose *Alces alces* (one host individual each) from Finland, and four specimens from the Siberian roe deer *Capreolus pygargus* (one host individual) from Russia.

Conspecificity of adults and metacestodes in various host species was confirmed using a partial nucleotide sequence (396 bp) of the mitochondrial cytochrome *c* oxidase subunit 1 (cox1) gene as previously described (Lavikainen et al. 2013). This region has been proved to be suitable for DNA barcoding of taeniids including the new species (Lavikainen et al. 2008, 2013). The sequences were compared with selected *cox1* sequences of *Taenia* spp. occurring in felids and/or cervids in the Holarctic region (8 species), and the phylogenetically related *Taenia regis*. The analyses were performed in MEGA7 (Tamura et al. 2013). The sequence set was aligned using ClustalW (Chenna et al. 2003). Pairwise divergences were calculated by Kimura 2–parameter (K2P) model (Kimura 1980) with a gamma setting 0.5. A phylogeny was constructed by the maximum likelihood method based on evolutionary model HKY+I (Hasegawa et al. 1985), as determined by the Bayesian information criterion. A maximum parsimony tree was used as the initial tree for the heuristic search, and the robustness of the phylogeny was tested by bootstrapping with 1000 replicates.

Adult cestodes were relaxed in water and fixed flat (without pressure) and preserved in 70–75% ethanol. Fragments of each specimen, representing various developmental stages, were stained with alum carmine, cleared in eugenol and mounted in Canada balsam. Hand–cut transverse sections of mature proglottids were prepared to determine the number of dorso–ventral testicular layers and the dorso–ventral position of terminal genital ducts with respect to the longitudinal ventral osmoregulatory canals and the nerve cord.

Cysticerci were fixed and preserved in 70–75% ethanol. The hook crowns extracted from cysticerci were mounted in Berlese’s medium for study. Only hooks aligned well in the horizontal plane were used for the morphometric analysis.

Five linear measurements, as defined by Gubányi (1995), were taken from large and small rostellar hooks (Table 1, Fig. 8). The measurements were defined using a longitudinal baseline drawn from the tip of the blade to the furthest point on the tip of the handle. TL (total length) is equal to the length of the baseline. TW (total width) is the distance between two longitudinal lines at the margins of the hook, drawn parallel to the baseline. PL (posterior length) is the distance from the tip of the handle to the tip of the guard. AL (anterior length) is the distance from the tip of the guard to the tip of the blade. GL (guard length) is the distance from the baseline to the tip of the guard, defined by a line drawn perpendicular to the baseline.

**Table 1.** Variation in measurements (µm) of large rostellar hooks in *Taenia lynciscapreoli* sp. n. Figures show the range with the mean in parentheses. TL, total length; TW, total width; PL, posterior length; AL, anterior length; GL, guard length (see Fig. 8).

Hosts, region	TL	TW	PL	AL	GL
*Lynx*, Finland (n=11)	168–228 (195.9)	78–94 (84.5)	114–162 (133.8)	76–97 (86.3)	42–54 (47.7)
*Lynx*, Russia (n=16)	214–231 (223.4)	79–96 (89.4)	138–162 (152.1)	87–101 (94.9)	40–59 (50.8)
*Lynx*, combined (n=27)	168–231 (212.2)	78–96 (87.4)	114–162 (144.7)	76–101 (91.4)	42–59 (49.5)
*Capreolus*, Finland (n=3)	213–222 (216.5)	85–92 (87.5)	136–153 (144.2)	95–98 (96.9)	48–56 (49.9)
*Capreolus*, Russia (n=15)	215–238 (230.7)	94–109 (103.4)	148–171 (162.7)	92–111 (104.3)	54–88 (65.6)
*Alces*, Finland (n=7)	213–230 (222.3)	82–97 (90.9)	145–162 (154.8)	86–100 (94.0)	46–60 (52.3)
Cervids, combined (n=25)	213–238 (225.9)	82–109 (97.2)	136–171 (157.2)	86–111 (100.3)	46–88 (59.2)
*Lynx* + cervids, combined (n=52)	168–238 (219.1)	78–109 (92.3)	114–171 (150.9)	76–111 (95.8)	40–88 (54.4)

The shape of the large rostellar hooks was compared by scaling a representative hook of each species to the same total length, and then aligning a pair of hooks using the outline of the junction between the blade and the guard as an anchor region. The form of the anchor region was almost invariable among the species considered here.

Type and voucher specimens have been deposited in the Finnish Museum of Natural History (MZH) and the Institute of Systematics and Ecology of Animals SB RAS, Novosibirsk, Russia (SVK).

## Results

### Genetic identification

**Figure 1.**
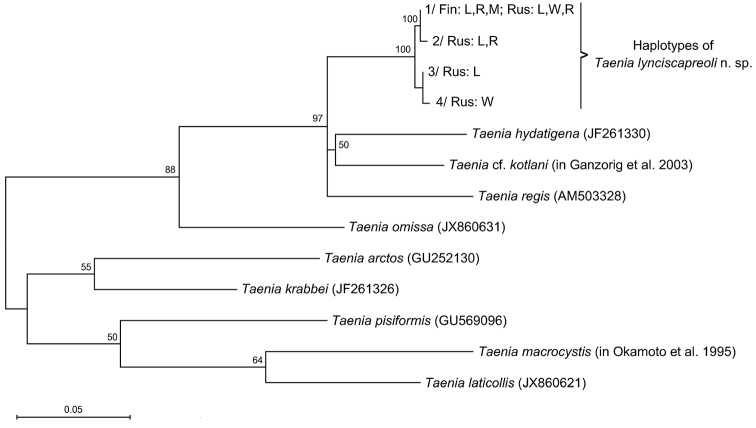
A phylogenetic tree of selected species of *Taenia* inferred from a 396 bp fragment of mitochondrial *cox1* gene by the maximum likelihood method. Bootstrap values >50% are shown. The scale bar represents the estimated number of substitutions per site. Accession numbers or references of the previously published sequences are in parentheses. The haplotypes of *Taenia lynciscapreoli* sp. n. are designated with numbers 1–4, and their geographical origins and hosts are indicated with abbreviations: Fin, Finland; Rus, Russia; L, lynx; W, wolf; R, European or Siberian roe deer; M, moose.

DNA sequences showed unambiguously that the specimens from various host species and regions represent the same species. Four *cox1* haplotypes were identified, the most common of which was identical with the *cox1* haplotype observed by Lavikainen et al. (2013) (GenBank accession number JX860629). The haplotypes formed a well–supported monophyletic entity (Fig. 1). Divergence values were 0.3-1.1% between the haplotypes of the new species, whereas between the new species and the most closely related species (*Taenia hydatigena*, *Taenia* cf. *kotlani* Murai, Gubányi & Sugar, 1993 and *Taenia regis*) divergences were clearly higher, 8.3-10.1%. New nucleotide sequence data of the new species is available in the DDBJ/EMBL/GenBank databases under the accession numbers KU324546–KU324548.

#### Taenia lynciscapreoli sp. n.

Taxon classificationAnimaliaCyclophyllideaTaeniidae

http://zoobank.org/559B4067-3FE2-4B35-86B6-03780ED73DDF

##### Material.

*Adult*. Type–material: Holotype MZH 127098 (five slides, including hand–cut transverse sections, and fragments in ethanol). Paratype MZH 127099 (three slides and fragments in ethanol), from the same host individual as the holotype.

Voucher material from *Lynx lynx*: MZH 127100 (three slides and fragments in ethanol), MZH 127101 (five slides and fragments in ethanol) and MZH 127102 (six slides and fragments in ethanol), Lohja, southern Finland; MZH 127105 (two slides) and MZH 127106 (three slides), Mikhailovskiy raion, Altai Rai, Russia.

Voucher material from *Canis lupus* (wolf): SVK-2265 and SVK-2581, Mikhailovskiy raion, Altai Rai, Russia.

Other museum specimens from *Lynx lynx* from Finland (in ethanol): MZH 123001, from Hyvinkää; MZH 123002, from Sauvo; MZH 123003 and 123004, from Mustasaari; MZH 123005, locality unknown.

Other records from *Lynx lynx*: Kolosovsky and Bolsheukovsky raions, Omskaya oblast’, Western Siberia, Russia (morphological identification), coll. Bykova, 2006 [identified as *Taenia pisiformis* (Bloch, 1780)].

##### Type host.

*Lynx lynx* Linnaeus, 1758, the Eurasian lynx. Other hosts: *Canis lupus* Linnaeus, 1758, the wolf.

##### Type locality.

Salo, Perniön Ylikulma (WGS 84: 60°16.948'N; 23°13.288'E), southern Finland.

Site. Small intestine.

##### Metacestode.

Host: European roe deer *Capreolus capreolus* (Finland), Siberian roe deer *Capreolus pygargus* (Russia) and Eurasian elk/moose *Alces alces* (Finland).

##### Voucher material.

MZH 127104 (two specimens in ethanol), *Alces alces* (calf), Hausjärvi, southern Finland; SVK-2344, SVK-2402 (in ethanol), SVK-2458, SVK-2395 (slides), *Capreolus pygargus*, Russian Far East.

##### Other museum specimens.

N16553, Museum of All–Russian K. I. Skryabin Scientific Research Institute of Helminthology (Moscow), *Capreolus pygargus*, Tuva Republic, Southern Siberia, Russia (identified as *Taenia hydatigena*).

Site. Liver and lungs.

##### Diagnosis.

Adults and metacestodes of *Taenia lynciscapreoli* sp. n. can be separated unambiguously from all other species of *Taenia* by the shape of their large rostellar hooks, particularly the characteristically short, wide and strongly curved blade. If the large rostellar hooks are missing in adults, *Taenia lynciscapreoli* may be separated from related species by a combination of morphological features of mature proglottids (see Discussion).

##### Description.

Measurements are in micrometres if not otherwise stated.

**Figure 2.**
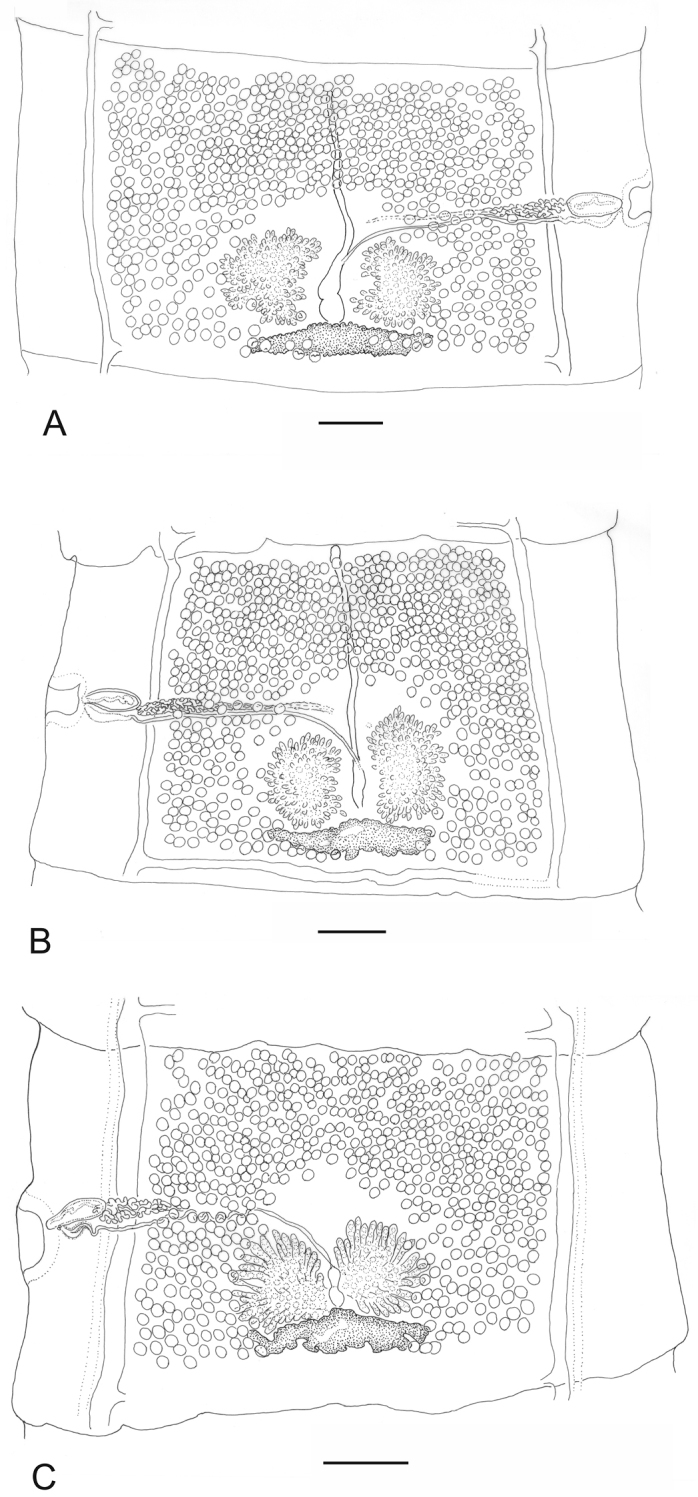
Mature proglottids of *Taenia lynciscapreoli* sp. n. from *Lynx lynx*. **A** holotype **B** paratype **C** voucher. Scale-bars: 500 μm (**A–B**); 300 μm (**C**).

**Figure 3.**
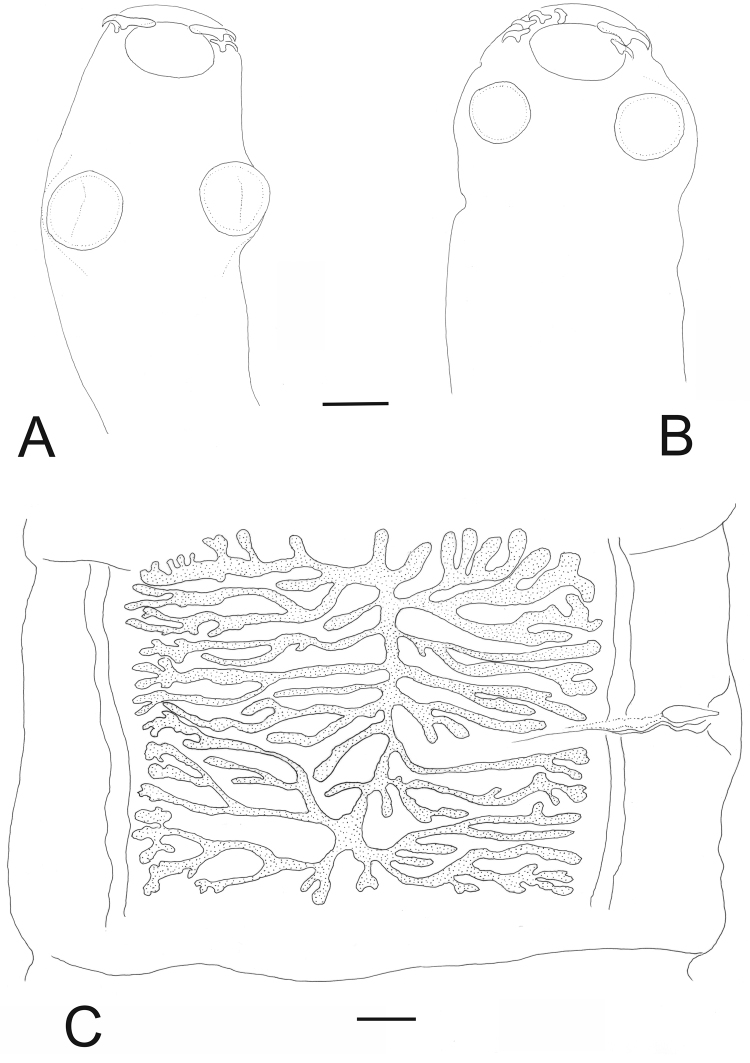
Scolex (**A**, **B**) and a pregravid proglottid with uterus (**C**) of *Taenia lynciscapreoli* sp. n. from *Lynx lynx*. **A, B** paratypes **C** voucher. Scale-bars: 200 μm (**A–B**); 500 μm (**C**).

**Figure 4.**
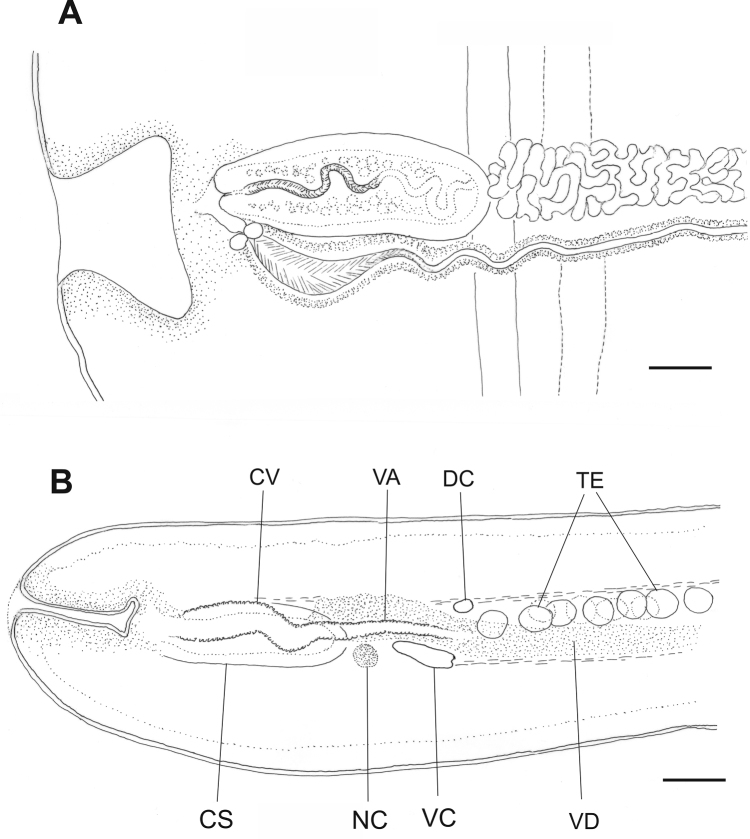
Terminal genital ducts of *Taenia lynciscapreoli* sp. n. (holotype) in whole mount (**A**) and in hand–cut transverse section (**B**). VC, ventral longitudinal osmoregulatory canal; DC, dorsal longitudinal osmoregulatory canal; NC, nerve cord; VA, vagina; CV, copulatory part of vagina; CS, cirrus sac; VD, vas deferens; TE, testes. Scale-bars: 100 μm.

**Figure 5.**
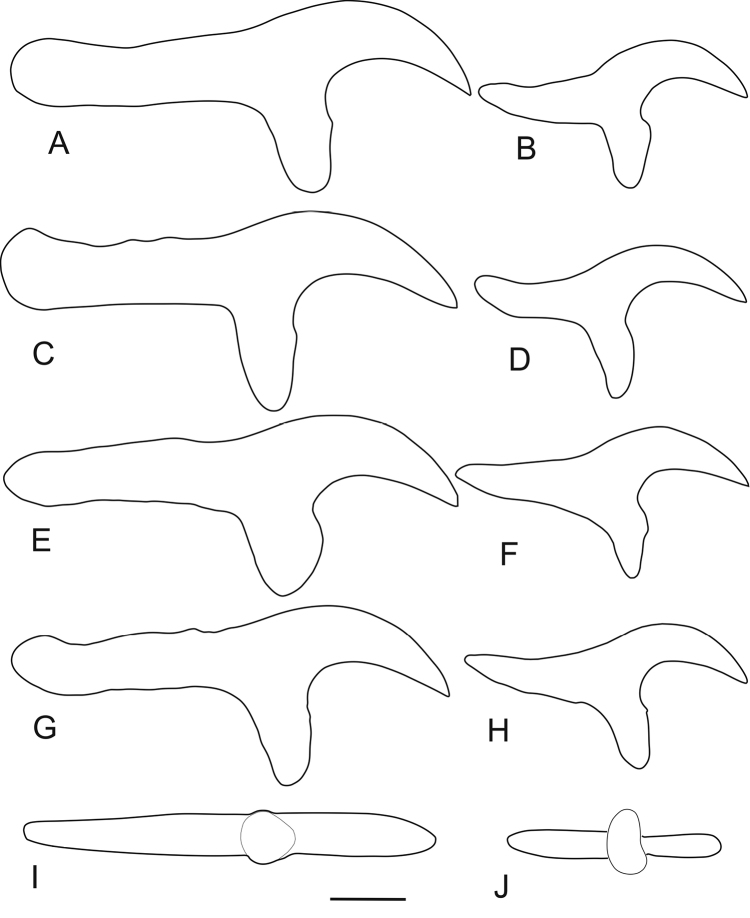
Outline drawings of large and small rostellar hooks of *Taenia lynciscapreoli* sp. n. from various host species. **A–H** side view **I–J** “ventral” view **A–B** *Lynx lynx* (holotype) **C–D** *Canis lupus* **E–F** *Capreolus capreolus* **G–J** *Alces alces*. Scale-bar: 50 μm.

**Figure 6.**
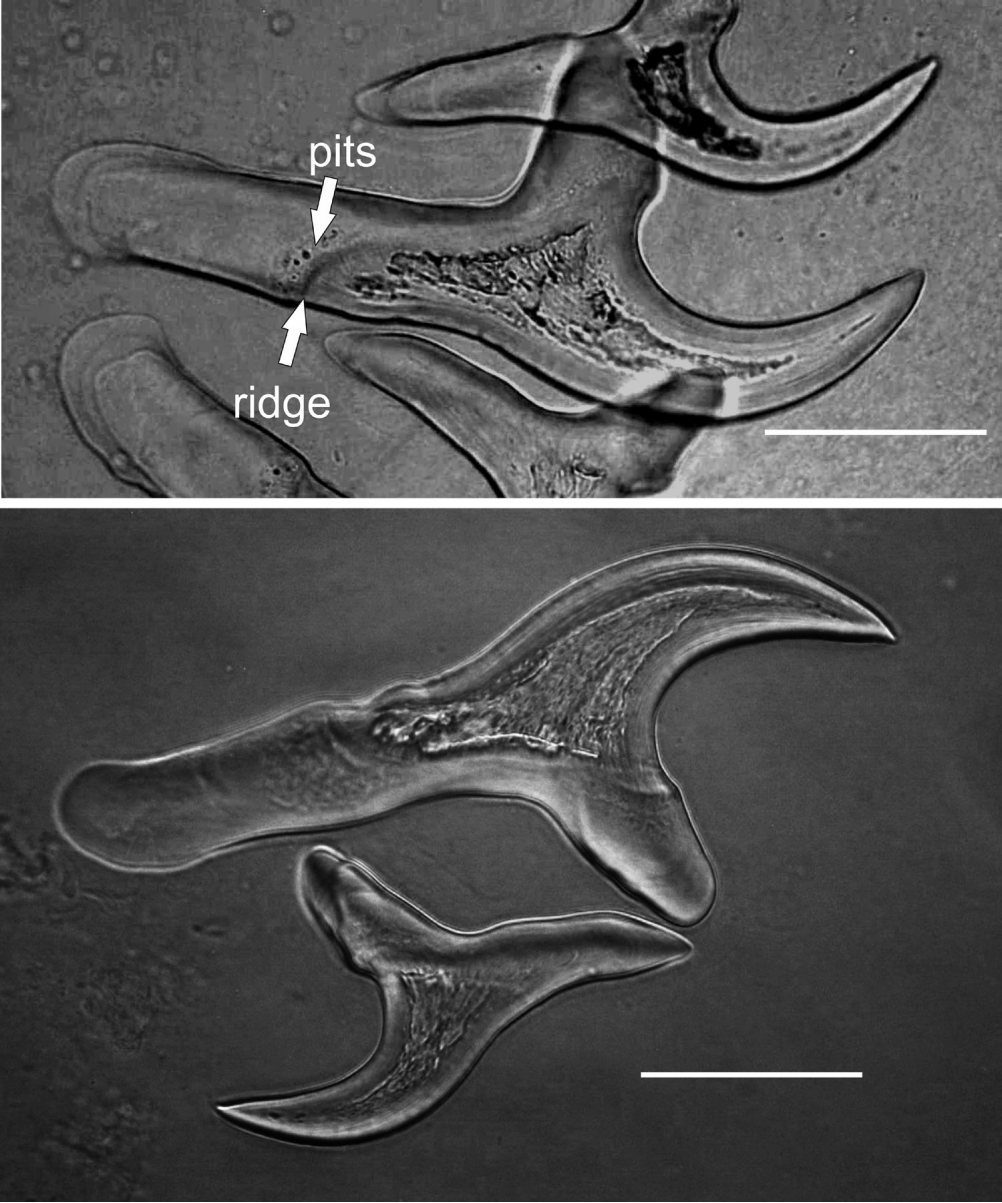
Large and small rostellar hooks of *Taenia lynciscapreoli* sp. n. from *Lynx lynx*, the higher picture showing the characteristic ridge and pits of large hooks. Scale-bars: 50 μm.

**Figure 7.**
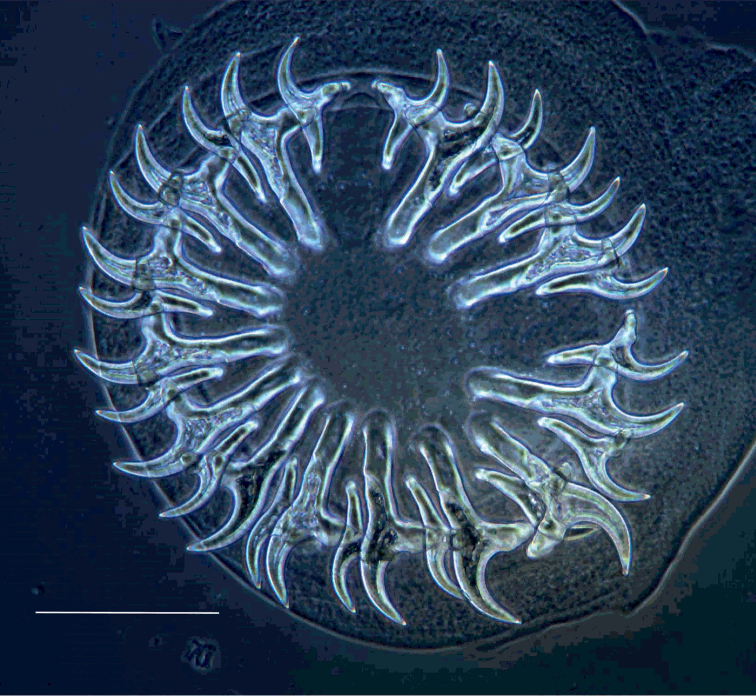
Hook crown of *Taenia lynciscapreoli* sp. n. from *Lynx lynx*. Scale-bar: 200 μm.

*Adult* (Figs 2–7; Table 1). Measurements of mature proglottids and scolex are based on specimens from Finland, and other measurements (external features, rostellar hooks, uterine branches, eggs) on combined material from Finland and Russia.

Medium–sized species of *Taenia*; length of fully gravid specimens 55–90 cm (n=4). Maximum width of strobila 5–7 mm (n=4). Scolex 1.1 mm (n=2) wide in specimens mounted in Berlese’s medium (BM), 0.85 mm (n=1) wide in specimens mounted in Canada balsam (CB). Maximum diameter of suckers 269–289 in BM (n=7), 213–255 in CB (n=4). Diameter of rostellum 375–425 in BM (n=2), 300–365 in CB (n=2); rostellum larger than suckers. Neck approximately as wide as scolex, of variable length.

Rostellum bearing two rows of hooks; rostellar armature usually incomplete in adult specimens. In combined material, length of large hooks 168–231 (mean=212.2, n=27) and length of small hooks 106–137 (mean=126.2, n=25). Total length and other dimensions of large hooks consistently smaller in specimens from Finland than in those from Siberia and Russian Far East. Large hooks characterized by long, thick and straight handle sometimes provided with apical bulge, relatively short, wide and strongly curved blade and prominent, usually slightly pointed guard. Border between hidden and exposed parts of large hooks marked with distinct oblique ridge. Margin of ridge provided with pits of various sizes at middle of handle; similar but less distinct pits sometimes present at guard portion of ridge.

Proglottids craspedote, but velum poorly developed. Mature proglottids 2.8–5.3 mm (mean=4.3 mm, n=15) wide and 2.0–3.4 mm (mean=2.6 mm, n=15) long, with length/width ratio of 1:1.2–2.6 (mean=1:1.7, n=15) in well–relaxed specimens. Proglottids becoming more elongate posteriorly; fully–gravid proglottids up to 14 mm long, with length/width ratio of 1:4.7.

Genital pores irregularly alternating, positioned in middle of lateral margin of proglottids. Genital atrium weak, usually not protruding, 238–425 (mean=302, n=12) wide at base and 144–264 (mean=186, n=12) deep. Ventral longitudinal osmoregulatory canals 34–110 (mean=75, n=13) wide in mature proglottids, up to 200 in postmature/pregravid proglottids; connected by narrower transverse canals. Dorsal osmoregulatory canals narrow (seen only in transverse sections), running medially to ventral longitudinal canals. Terminal genital ducts positioned between dorsal and ventral longitudinal osmoregulatory canal and dorsal to nerve–cord.

Testes 591–725 (mean=653, n=5) in number, 80–130 in largest diameter, positioned primarily in one dorso–ventral layer. Testicular field widely confluent anteriorly and occupying all parts of median field lacking female organs, except small well–defined region anterior to ovary. Continuous posterior testicular field absent, but sometimes individuals testes positioned posterior to or overlapping vitellarium. Antero–poral testicular field longitudinally as long as postero–poral field (as separated by vas deferens). Testicular field separated from ventral osmoregulatory canals by distinct free space laterally, anteriorly and posteriorly. Cirrus–sac elongate, 340–425 (mean=382, n=11) long and 153–179 (mean=166, n=11) wide in mature proglottids, usually not extending to longitudinal ventral canal; muscle layers of cirrus–sac well–developed. Distal part of ductus cirri armed with delicate hair–like structures. Vas deferens forming few irregular loops inside cirrus–sac, prominently convoluted outside cirrus–sac.

Ovary bilobed, 98–172 (mean=150, n=15) wide and 57–103 (mean=84, n=15) long; lobes of roughly equal size, but antiporal lobe extending slightly more anteriad than poral lobe; ovary does not reach midline of proglottid longitudinally. Vitellarium distinctly elongated transversely, 80–145 (mean=126, n=15) wide and 19–41 (mean=31, n=12) long, slightly narrower than ovary; lateral extremities usually pointed. Vagina opens posterior to male pore, provided by distinct sphincter ca. 5 from distal end of vagina; sphincter ca. 3 long and 6 wide; sphincter sometimes absent or incomplete (present on one side of vagina only). Copulatory part of vagina shorter than cirrus sac, thick–walled, distinctly widened, curved posteriorly; maximum width of copulatory part 94–111 (mean=106, n=10). Proximal vagina narrow, of uniform width, runs posterior to vas deferens, usually slightly undulating, rarely looped. Lumen of vagina lined with delicate hair–like structures almost throughout its length; hairs particularly long in widened copulatory part. Prior to joining seminal receptacle, vagina forms differentiated region, 10–12 long, with tapered lumen lacking hairs. Sperm–filled seminal receptacle elongate, 9–17 (mean=12.4, n=15) long. Mehlis’ gland spherical, 18–22 (mean=19.6, n=11) in diameter. Uterus in pre–gravid and early gravid proglottids with 8–11 primary branches on each side, often with secondary and tertiary bifurcations; lateral branches not reaching ventral osmoregulatory canal; terminal branches usually with multiple anterior or posterior sacculations. Eggs spherical or subspherical, with maximum diameter of 34–39 (mean=36.8, n=26) in whole–mounts. Outer egg shell thick (4.0–4.5), distinctly two–layered.

*Metacestode* (Fig. 5, Table 1). External features of metacestodes are based on specimens from Finland, and measurements of rostellar hooks on combined material from Finland and Russia (Table 1).

Metacestode is cysticercus. Ethanol–fixed cysticerci with fully–developed rostellar hooks 3–14 mm long and 2–5 mm wide; larger cysticerci with elongate or sac–like posterior bladder and, in one case, with short (8 mm) strobila between bladder and scolex region. Rostellum armed with 30–34 (mean=32.0, n=7) hooks forming two rows. Large hooks 213–238 (mean=225.9, n=27) and small hooks 123–145 (mean=136.7, n=23) long. Average hook dimensions are consistently smaller in specimens from Finland than in specimens from Siberia and Russian Far East. Rostellar hooks of metacestodes are similar in shape to those of adult cestodes.

##### Distribution.

Eurasia, from Finland to Russian Far East.

##### Etymology.

The specific epithet refers to the main definitive and intermediate hosts of the new species.

## Discussion

### Main morphological differences between *Taenia lynciscapreoli* sp. n. and related species

*Taenia lynciscapreoli* sp. n. is compared with all congeneric species parasitizing felids (definitive hosts) or cervids (intermediate hosts) in the Holarctic region (12 species), and also with the phylogenetically closely related *Taenia regis* (see Lavikainen et al. 2013). When compared with the new species (Table 2), *Taenia arctos*, *Taenia hydatigena*, *Taenia kotlani*, *Taenia pisiformis*, *Taenia krabbei* Moniez, 1879 and *Taenia parenchymatosa* Pushmenkov, 1945 showed overlapping numbers and/or lengths of rostellar hooks, and were therefore selected for comparison of the shape of the rostellar hooks. In addition, *Taenia ingwei* Ortlepp, 1938, a parasite of *Panthera pardus* in Africa, was selected for hook shape comparison, because it shows highest overlap in the number and length of rostellar hooks among *Taenia* spp. of African/Asian felids, when compared with *Taenia lynciscapreoli*.

**Table 2.** Host species and characteristics of rostellar hooks of *Taenia* spp. compared with *Taenia lynciscapreoli* sp. n., based on Loos-Frank (2000) and Haukisalmi et al. (2011). Hook characteristics showing highest overlap with those of *Taenia lynciscapreoli* sp. n. indicated in bold.

*Taenia* spp.	Definitive hosts	Intermediate hosts	Geographic distribution	Number of hooks	Large hooks, length	Small hooks, length
***Taenia lynciscapreoli* sp. n.**	**felids (*Lynx*)**	**cervids (*Capreolus*)**	**Eurasia**	**30–34**	**168–238**	**106–145**
*Taenia arctos* Haukisalmi, Lavikainen, Laaksonen & Meri, 2011	bears (*Ursus*)	cervids (*Alces*)		**22–36**	**153–180**	**96–130**
*Taenia hydatigena* Pallas, 1766	canids	cervids and other ruminants	worldwide	**28–44**	**169–235**	**110–168**
*Taenia ingwei* Ortlepp, 1938	felids (*Panthera*)	unknown	Africa	**32–34**	**197–202**	148–151
*Taenia kotlani* Murai, Gubanyi & Sugar, 1993	unknown, probably felids (*Panthera*)	bovids (*Capra*)	Central Asia	**30–36**	**187–218**	**118–143**
*Taenia* cf. *kotlani* of Ganzorig et al. (2003)^†^	*Panthera*	unknown, probably cervids	Central Asia	**30–35**	**190–209**	**127–144**
*Taenia krabbei* Moniez, 1879	canids	cervids and other ruminants	Holarctic region	**22–36**	**137–195**	**84–141**
*Taenia laticollis* Rudolphi, 1819	felids (*Lynx*)	lagomorphs	Eurasia	58–66	370–420	150–247
*Taenia macrocystis* (Diesing, 1850)	felids (*Lynx*, *Leopardus*, *Puma*)	lagomorphs	America, Asia	54–74	297–430	180–247
*Taenia omissa* Lühe, 1910	felids (*Puma*, *Leopardus*)	cervids (*Odocoileus*)	America	38–44	223–297	165–223
*Taenia parenchymatosa* Pushmenkov, 1945	canids	cervids	Russia	**30–34**	**210–240**	**124–160**
*Taenia parenchymatosa* of Murai et al. 1993^‡^	felids (*Lynx*)	cervids (*Capreolus*)	Siberia	**27–34**	**195–234**	**118–149**
*Taenia pisiformis* (Bloch, 1780)	canids, occasionally felids including *Lynx*	lagomorphs	worldwide	34–46	**220–300**	**114–177**
*Taenia pseudolaticollis* Verster, 1969	felids (*Lynx*, *Leopardus*)	unknown (probably lagomorphs)	America	38–42	352–415	214–240
*Taenia regis* Baer, 1923	felids (*Panthera*)	bovids (antelopes), suids (*Phacocoerus*)	Africa	32–49	223–273	142–199
*Taenia rileyi* Loewen, 1929	felids (*Lynx*, *Puma*)	rodents	America	36–46	238–258	145–198

† *Taenia* cf. *kotlani* of Ganzorig et al. (2003) is considered here to be conspecific with *Taenia kotlani* Murai, Gubanyi & Sugar, 1993.

‡ *Taenia parenchymatosa* of Murai et al. (1993) is considered here to be conspecific with *Taenia lynciscapreoli* sp. n.

**Figure 8.**
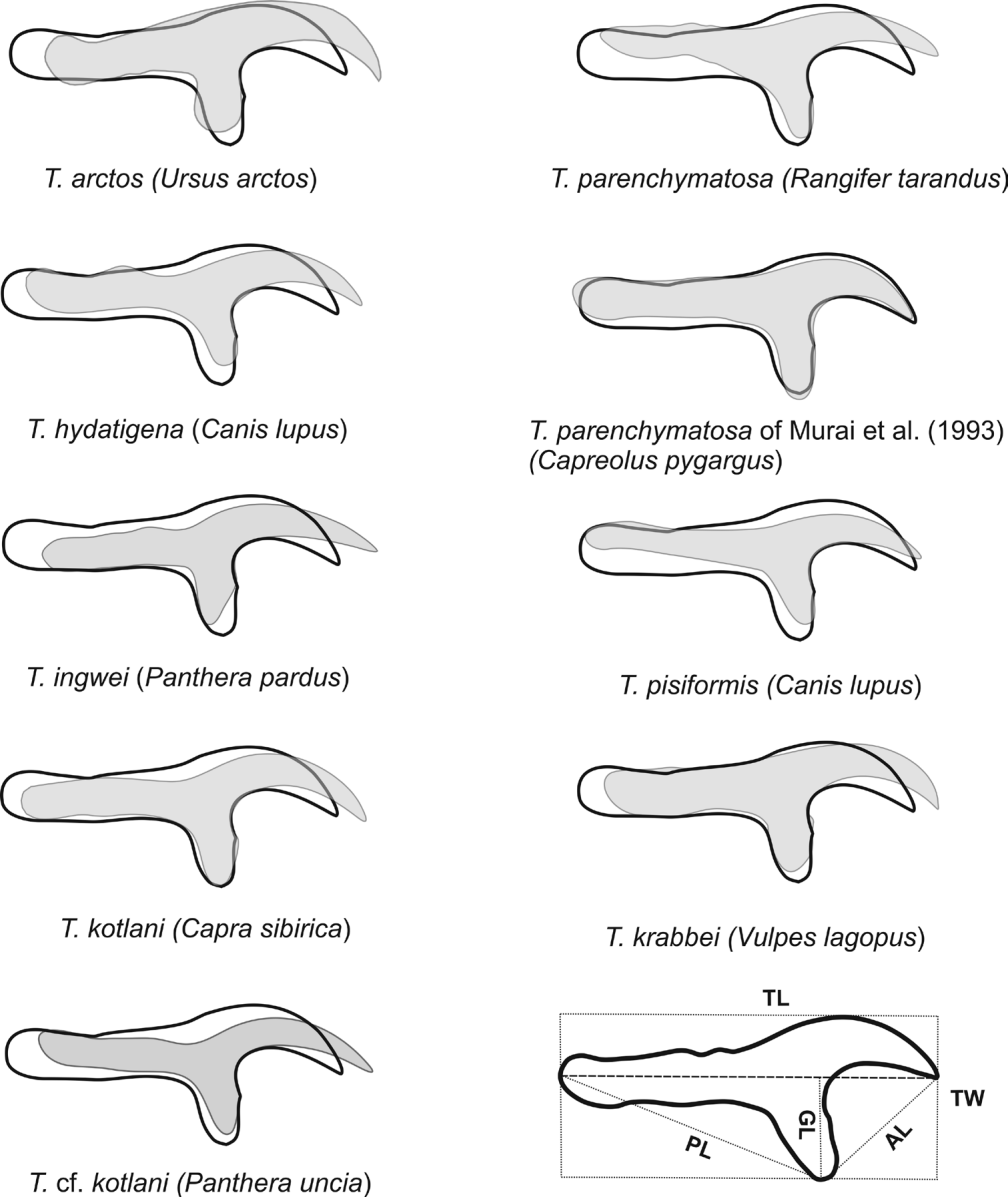
Pairwise comparisons of the shape of the large rostellar hooks in *Taenia lynciscapreoli* sp. n. and related species, using the junction between the blade and the guard as an anchor region for alignment. The hook of *Taenia lynciscapreoli* sp. n. is indicated by a black outline. A legend for measurements taken from the large hooks of the new species (Table 1) is also shown.

When aligned using the outline of the junction between the blade and the guard, the large rostellar hooks of *Taenia lynciscapreoli* have a shorter blade and longer handle, and a wider and more strongly curved blade than those of the other species, with the partial exception of *Taenia pisiformis* (Fig. 8). The latter species can be distinguished from *Taenia lynciscapreoli* by its more numerous rostellar hooks and the narrower and less curved blade of the large hooks.

Interspecific differences in the morphology of mature proglottids between *Taenia lynciscapreoli* and the species showing the highest overlap in hook characteristics are listed in Table 3. All species compared, with the exception of *Taenia ingwei*, can be unambiguously separated from *Taenia lynciscapreoli*. The difference in the shape of the large hooks appears to be the only reliable way to distinguish *Taenia ingwei* and *Taenia lynciscapreoli*. Additional differences are, however, expected to be found, if the morphology of the mature proglottids of *Taenia ingwei* were examined in greater detail. It should be noted that there is a disagreement concerning the distribution of testes between the descriptions of *Taenia ingwei* by Ortlepp (1938) and Verster (1969). The former specifically states that there are no testes posterior to vitellarium, which is shown in his illustration, whereas the latter states that the testicular field is “confluent dorso–posteriorly to the vitellarium”. Either this feature is variable in *Taenia ingwei*, which is the case in *Taenia lynciscapreoli*, or the redescription of Verster (1969) is composite (her description was based on the type specimens and “additional adults from the same host and locality”).

**Table 3.** Comparison of morphological features of mature proglottids in *Taenia lynciscapreoli* sp. n. and species showing highest overlap in the number and length of rostellar hooks. There is no adequate description for the morphology of the adult of *Taenia kotlani*. Based on Loos-Frank (2000) and Haukisalmi et al. (2011).

Taenia spp.	Vaginal sphincter	Longitudinal extent of ovary	Antiporal lobe of ovary distinctly larger than poral lobe	Free space around testes	Length of poral testicular fields	Width of anterior testicular field	Number of testicular layers
*Taenia lynciscapreoli* sp. n.	+	< midline	–	+	A = P^†^	wide	1
*Taenia arctos*	+	> midline	+	–	A = P	wide	2–3
*Taenia hydatigena*	–	< midline	+	–	A > P	wide	1
*Taenia ingwei*	+	≤ midline	–	?	A = P	wide	1
*Taenia krabbei*	+	< midline	+	+	A > P	wide	1–2
*Taenia parenchymatosa*	+	= midline	–	+	A < P	narrow	?
*Taenia pisiformis*	–	< midline	+	–	A > P	wide	2–4

† A, antero–poral testicular field; P, postero–poral testicular field (as separated by terminal genital ducts).

### Rostellar hooks

Gubányi (1995) applied multivariate morphometrics for rostellar hooks of 18 species of *Taenia* s.l., and concluded that “*Taenia parenchymatosa* and *Taenia laticollis* can be very well differentiated (100%) from the other species by the small and large hooks”. In addition to *Taenia parenchymatosa* of Murai et al. (1993) and *Taenia laticollis* Rudolphi, 1819, the analysis of Gubányi (1995) included *Taenia hydatigena*, *Taenia kotlani*, *Taenia pisiformis* (Bloch, 1780) and *Taenia regis*, all of which are included in our interspecific comparison (Table 2), but also two additional species from African felids (*Taenia acinomyxi* Ortlepp, 1938 and *Taenia selousi* Mettrick, 1963). As shown below, *Taenia parenchymatosa* of Murai et al. (1993) and Gubányi (1995) from *Capreolus pygargus* from Siberia is almost certainly conspecific with *Taenia lynciscapreoli*, and the results of Gubányi (1995) therefore provide further support for the status of the new species as a morphologically distinct entity.

In practice, the identification of *Taenia lynciscapreoli* based on rostellar hooks is straightforward; the new species has shorter hooks than other congeneric species parasitizing felids in the Holarctic region, with the possible exception of *Taenia kotlani*, the definitive host of which is unknown. The identification of metacestodes parasitizing cervids is slightly more challenging, but the present comparison shows that the unique shape of the large hooks of *Taenia lynciscapreoli*, particularly the short, wide and strongly curved blade, separates it from other species with rostellar hooks of similar length. If properly compared, the characteristic shape of the large hooks of *Taenia lynciscapreoli* also serves to separate it from all other species of *Taenia*, including those not compared here with the new species (see Gubányi 1995 and the Global Cestode Database; Caira et al. 2012).

Total length has often been the only feature used to characterize the rostellar hooks of *Taenia* spp., although it may be assumed that the shape of the hooks is a taxonomically more informative feature. Interspecific differences in the shape of rostellar hooks have been analysed using multivariate morphometrics (Gubányi 1995, 1996), but such an approach in somewhat unpractical for taxonomical purposes. A more straightforward and practical approach, as applied here, is to scale (large) rostellar hooks to the same total length and align them using an “anchor region” that shows limited variation among species. In this way it is easy to visualize interspecific differences in the shape and proportions of rostellar hooks. Such shape differences should also be easy to quantify, for example, by measuring the overlap between a pair of aligned hooks. This method is naturally most useful when comparing tapeworm species that show overlapping hook dimensions. Intraspecific comparisons of hook shape in *Taenia lynciscapreoli*, *Taenia arctos*, *Taenia hydatigena*, *Taenia krabbei*, *Taenia laticollis*, *Taenia martis* (Zeder, 1803) and *Taenia polyacantha* Leuckart, 1856 show that the shape of the blade of the large hooks is very constant within each species, but the shape of the handle and guard are more variable (not shown).

The large hooks of the cestode from *Capreolus pygargus* from Siberia, identified by Murai et al. (1993) as *Taenia parenchymatosa*, match well with the hook shape of *Taenia lynciscapreoli* (Fig. 8). Similar hook shape and strong overlap in hook number and length suggest that the *Taenia parenchymatosa* of Murai et al. (1993) actually represents *Taenia lynciscapreoli*. Congeneric intermediate hosts (*Capreolus* spp.) support their conspecificity. The hook comparison also suggests that that *Taenia* cf. *kotlani* from the snow leopard (Ganzorig et al. 2003) is conspecific with *Taenia kotlani* from the Siberian ibex *Capra sibirica* (Murai et al. 1993).

### Phylogenetics

Besides *Taenia lynciscapreoli*, there are published DNA sequence data for five species of *Taenia* s.s. parasitizing felids, i.e. *Taenia* cf. *kotlani* (Ganzorig et al. 2003), *Taenia laticollis* (Lavikainen et al. 2013, Nakao et al. 2013), *Taenia macrocystis* (Diesing, 1850) (Okamoto et al. 1995), *Taenia omissa* Lühe, 1910 (Lavikainen et al. 2013, Gomez–Puerta et al., published only in GenBank), and *Taenia regis* (Zhang et al. 2007). The present and previously published phylogenetic analyses show unambiguously that none of these can be conspecific with *Taenia lynciscapreoli*. Although *Taenia lynciscapreoli* groups with *Taenia* cf. *kotlani*, *Taenia regis* and *Taenia hydatigena*, the latter of which uses canids as definitive hosts, the genetic distances between these species are at an interspecific level (Zhang et al. 2014). The phylogenetic analysis by Lavikainen (2014) included an additional species from felids, i.e. *Taenia rileyi* Loewen, 1929 (*Lynx* and *Puma*, Nearctic; unpublished sequence), which formed a separate clade with *Taenia omissa* (Fig. 3 in Lavikainen 2014), clearly distinct from *Taenia lynciscapreoli*.

*Taenia lynciscapreoli* was not compared here morphologically with *Taenia* spp. parasitizing felids in Africa and Asia, because, according to present knowledge, their fauna is separate from the corresponding fauna in the Holarctic region. However, *Taenia regis*, a parasite of the lion in Africa, is included in the present comparison, because it is phylogenetically related to *Taenia lynciscapreoli*. It is possible that there are more extensive phylogenetic connections between *Taenia* spp. of Holarctic and southern felids, but there are no published DNA sequence data for species of *Taenia* other than *Taenia regis* parasitizing felids in Africa or Asia. However, our unpublished data suggest that *Taenia gonyamai* Ortlepp, 1938 and *Taenia selousi* Mettrick, 1963, parasites of felids in Africa, are phylogenetically distinct entities and therefore not conspecific with *Taenia lynciscapreoli*.

A group of taeniid cestodes, including two species parasitizing felids [*Hydatigera taeniaeformis* (Batsch, 1786) and *Hydatigera krepkogorski* Schulz & Landa, 1934], was recently shown to form a distinct clade by molecular phylogenetic methods, and therefore proposed to represent the resurrected genus *Hydatigera* Lamarck, 1816 (see Nakao et al. 2013). *Hydatigera* spp. can be easily distinguished from *Taenia* spp. by their long rostellar hooks and a strobilocercus–type metacestode.

### Life cycle and host specificity

The existing data on *Taenia lynciscapreoli* strongly suggests that it uses specifically the lynx and the roe deer as definitive and intermediate hosts, respectively. Being small cervids, roe deer are optimal and, where available, preferred prey items for the lynx (Pulliainen 1981, Jedrzejewski et al. 1993, Odden et al. 2006).

The lynx and the roe deer have almost continent–wide, overlapping distributions in Eurasia, although the latter host is represented by two allopatric species (*Capreolus capreolus* and *Capreolus pygargus*). However, the distribution of the Eurasian lynx extends further north than the distribution of the roe deer, and, if the occurrence of the parasite is dependent on the presence of both primary hosts, we would expect to find the parasite in the lynx only in regions inhabited by the roe deer. This seems to be case in Finland, as Lavikainen et al. (2013a) found *Taenia lynciscapreoli* (referred to as “*Taenia* sp.”) in lynx from southern and western Finland, where the roe deer is abundant, but not from the more northern and eastern parts of the country where the roe deer is absent or sporadic. In accordance, the present new findings of *Taenia lynciscapreoli* in the lynx are from southernmost Finland. Similarly, the present findings of *Taenia lynciscapreoli* in Russia are located within the range of *Capreolus pygargus*.

However, despite the basically strict host–specificity, accidental infections of other definitive host species are likely to occur, especially with unrelated predators utilizing same intermediate host species. The present finding of *Taenia lynciscapreoli* in the wolf, confirmed by molecular methods, shows that such spill–over does happen. In this case the obvious explanation is that wolves prey on roe deer, the primary intermediate host of *Taenia lynciscapreoli*.

The finding of *Taenia lynciscapreoli* in the Eurasian moose calf, confirmed by molecular methods, shows that the new species is able to infect also cervids other than the roe deer. Although the lynx may succeed in killing a moose calf (Birkeland and Myrberget 1980), the moose is an exceptional prey species and thus cannot be involved in the normal transmission of the parasite.

It may be that infections of larger cervids (*Alces*, *Cervus*) by the metacestodes of *Taenia lynciscapreoli* occur only in regions where there exists a transmission cycle between the lynx and the roe deer.

### Possible misidentifications of *Taenia lynciscapreoli*

Because *Taenia lynciscapreoli* is evidently a predictable, wide–spread component in the tapeworm fauna of the lynx and the roe deer, it is probably represented in some previous studies, but has been misidentified or remained unidentified.

A survey of helminths of the lynx in Estonia (Valdmann et al. 2004) showed a rather unexpected result for *Taenia* spp., because every lynx (n=37) was infected with *Taenia pisiformis* (besides the less prevalent *Taenia laticollis* and *Taenia hydatigena*). *Taenia pisiformis* is typically a parasite of canids, particularly the wolf and the dog, with lagomorphs serving as the primary intermediate hosts (Loos-Frank 2000). An experimental study by Beveridge and Rickard (1975) showed that the domestic cat is not a suitable definitive host for *Taenia pisiformis*, because the worms developed slowly and the infections were lost before the worms became gravid. However, *Taenia pisiformis* has been reported several times also from other felids, particularly from the wild and domestic cats (*Felis catus*) (see the Host–parasite database of the Natural History Museum, London; Gibson et al. 2005), but also from the Iberian lynx *Lynx pardinus* (see Rodriguez and Carbonell 1998, Torres et al. 1998) and the North American *Lynx canadensis* (see Zyll de Jong 1966, Smith et al. 1986) and *Lynx rufus* (see Tiekotter 1985).

The identification of *Taenia* spp. by Valdmann et al. (2004) was based primarily on the total length of rostellar hooks, although “genital sacs” were also considered. The rostellar hooks of *Taenia pisiformis* are somewhat longer than those of *Taenia lynciscapreoli*, but still overlapping (Table 2), and both species can be classified as “short–hooked” among *Taenia* spp. In addition, the relative lengths of the handle and the blade of the large rostellar hooks are very similar in *Taenia pisiformis* and *Taenia lynciscapreoli*, although the latter has a wider and more curved blade (Fig. 8). Based on the apparent similarity of hook characteristics in these species, we assume that *Taenia pisiformis* of Valdmann et al. (2004) was actually *Taenia lynciscapreoli*. These two species could be separated by the number of rostellar hooks (higher in *Taenia pisiformis*), but rostellar hooks, particularly the long ones, are easily lost in adult specimens. The roe deer is abundant is Estonia and dominates in the diet of lynx (Valdmann et al. 2005), which should enhance the transmission of *Taenia lynciscapreoli* and explain its high prevalence.

Rodriguez and Carbonell (1998) reported *Taenia pisiformis* as a relatively common parasite of *Lynx pardinus* in south–central Spain, although they could not find its metacestodes in lagomorphs. Torres et al. (1998) also reported *Taenia pisiformis* in *Lynx pardinus* from the same region, but at a lower prevalence. The Iberian lynx examined in these studies originated from the Montes des Toledo region, where it co–occurs with the roe deer. The authors do not explain how the tapeworms from the Iberian lynx were identified, but it is again possible that *Taenia pisiformis* of Rodriguez and Carbonell (1998) and Torres et al. (1998) was actually *Taenia lynciscapreoli*. However, the reports of *Taenia pisiformis* in *Lynx canadensis* most probably do not represent *Taenia lynciscapreoli*, because there are no roe deer in North America.

It is obvious that some of the existing reports of *Taenia* metacestodes in roe deer, particularly in regions where it co–occurs with lynx, may also be *Taenia lynciscapreoli*. Three other valid species of *Taenia* using cervids as intermediate hosts in Eurasia, i.e. *Taenia krabbei* (including the probable junior synonym *Taenia cervi* Christiansen, 1931), *Taenia hydatigena* and *Taenia parenchymatosa*, may all be confused with *Taenia lynciscapreoli* because of overlapping hook number and dimensions (as shown above, *Taenia parenchymatosa* of Murai et al. 1993 from *Capreolus pygargus* is actually *Taenia lynciscapreoli*). The new species could be easily identified by the shape of the hooks, but rostellar hooks have seldom been described in reports concerning *Taenia* metacestodes of roe deer and other cervids. With the exception of Murai et al. (1993), the existing reports on *Taenia* metacestodes of cervids with hook illustrations (Christiansen 1931, Brzheskii 1963, Murai and Sugár 1979, Priemer et al. 2002) do not, however, include *Taenia lynciscapreoli*.

## Supplementary Material

XML Treatment for Taenia lynciscapreoli
